# Subgrouping germinal center-derived B-cell lymphomas based on machine learning-deduced DNA methylation modules

**DOI:** 10.1038/s41375-025-02533-6

**Published:** 2025-03-10

**Authors:** Selina Glaser, Anja Fischer, Juan Emilio Martínez-Manjón, Cristina López, Helene Kretzmer, Birgit Burkhardt, Daniel Hübschmann, Michael Hummel, Wolfram Klapper, Julia Kolarova, Markus Kreuz, German Ott, Bernhard Radlwimmer, Maciej Rosolowski, Matthias Schlesner, Andreas Rosenwald, Stephan Stilgenbauer, Rabea Wagener, Igor Zwir, Lorenz Trümper, Ralf Küppers, Peter Lichter, Ole Ammerpohl, Coral del Val, Reiner Siebert, on behalf of ICGC MMML-Seq consortium

**Affiliations:** 1 Institute of Human Genetics, Ulm University and Ulm University Medical Center, Ulm, Germany; 2 Department of Computer Science and Artificial Intelligence, Andalusian Research Institute in Data Science and Computational Intelligence (DaSCI), University of Granada, Granada, Spain; 3 Institute of Human Genetics, Christian-Albrechts-University, Kiel, Germany; 4 Institut d’Investigacions Biomèdiques August Pi i Sunyer, Barcelona, Spain; 5 Hematopathology Section, Pathology Department, Hospital Clínic de Barcelona, Barcelona, Spain; 6 Interdisciplinary Center for Bioinformatics, University of Leipzig, Leipzig, Germany; 7 Bioinformatics Group, Department of Computer, University of Leipzig, Leipzig, Germany; 8 Transcriptome Bioinformatics, LIFE Research Center for Civilization Diseases, University of Leipzig, Leipzig, Germany; 9 Department of Genome Regulation, Max Planck Institute for Molecular Genetics, Berlin, Germany; 10 Pediatric Hematology and Oncology, University Hospital Münster, Münster, Germany; 11 Innovation and Service Unit for Bioinformatics and Precision Medicine, German Cancer Research Center (DKFZ), Heidelberg, Germany; 12 Computational Oncology Group, Molecular Precision Oncology Program (MPOP), National Center for Tumor Diseases (NCT) Heidelberg and DKFZ, Heidelberg, Germany; 13 Pattern Recognition and Digital Medicine Group, Heidelberg Institute for Stem Cell Technology and Experimental Medicine (HI-STEM), Heidelberg, Germany; 14 German Cancer Consortium (DKTK), Heidelberg, Germany; 15 CharitéCenter for Biomedicine (CC4), Charité – University Medicine Berlin, Berlin, Germany; 16 Hematopathology Section, Institute of Pathology, Christian-Albrechts-University, Kiel, Germany; 17 Institute for Medical Informatics Statistics and Epidemiology, University of Leipzig, Leipzig, Germany; 18 Department of Clinical Pathology, Robert-Bosch Krankenhaus, and Dr. Margarete Fischer-Bosch Institute of Clinical Pharmacology, Stuttgart, Germany; 19 Division of Molecular Genetics, German Cancer Research Center (DKFZ), Heidelberg, Germany; 20 Biomedical Informatics, Data Mining and Data Analytics, Faculty of Applied Computer Science and Medical Faculty, University of Augsburg, Augsburg, Germany; 21 Institute of Pathology, University of Würzburg, Würzburg, Germany; 22 Department of Internal Medicine III, University of Ulm, Ulm, Germany; 23 Instituto de Investigación Biosanitaria ibs.GRANADA, Complejo Hospitales Universitarios de Granada/Universidad de Granada, Granada, Spain; 24 Department of Hematology and Oncology, Georg August University Göttingen, Göttingen, Germany; 25 Institute of Cell Biology (Cancer Research), University of Duisburg-Essen, Medical School, Essen, Germany

**Keywords:** B-cell lymphoma, Cancer epigenetics

## To the Editor:

Follicular lymphoma (FL) and diffuse large B-cell lymphoma (DLBCL) are the most common lymphomas, both exhibiting features of germinal center-derived B-cells (gcBCs). DLBCL can arise de novo or through transformation of a low-grade lymphoma like FL. FL and DLBCL are heterogeneous with regard to clinical outcome as well as morphologic, immunophenotypic, genetic, transcriptomic and other biological aspects [1]. Besides classic FL harboring the *IGH*::*BCL2* translocation, several subtypes with varying morphologic and genetic features exist [1]. Regarding DLBCL, cell-of-origin (COO) gene expression signatures classify them into germinal center B-cell-like (GCB) and activated B-cell-like (ABC) subtypes [2]. Additionally, several groups have identified clusters of DLBCL based on the mutational landscape and linked to clinical outcome [3–8].

**Fig. 1:**
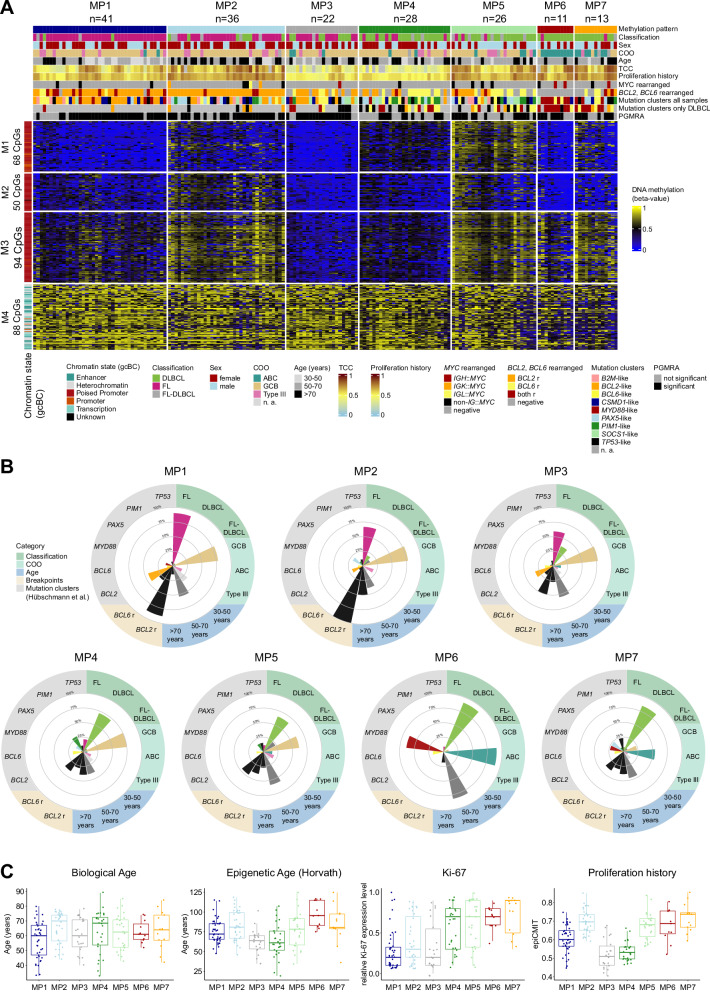
DNA methylation profiling and characterization of germinal center-derived B-cell lymphomas. Using PGMRA on DNA methylation array data from germinal center-derived B-cell lymphomas (FL, DLBCL, FL-DLBCL), we identified 300 CpGs, which were subsequently organized into four modules (M1-M4) and seven methylation patterns (MPs) through k-means clustering. **A** Heatmap depicting DNA methylation levels of the 300 CpGs differentiating seven MPs (MP1-7). Sample features are annotated at the top of the heatmap. The mutation clusters were calculated for the entire dataset and DLBCL cases separately as described by Hübschmann et al. [8]. The TCC was calculated based on WGS data. Rows represent individual CpGs, and columns represent samples. CpG sites and samples are organized according to the k-mean clustering. **B** Radar plots illustrate the key defining features of each MP, including lymphoma classification, patient age, cell-of-origin (COO), *BCL2* or *BCL6* rearrangements, and mutational clusters identified by Hübschmann et al. **C** Boxplots display the distribution of biological age, Horvath’s epigenetic age, Ki-67 expression, and proliferation history (based on the epiCMIT package) across MPs. For statistical testing a pairwise Wilcoxon rank sum test with Bonferroni correction was applied (see Supplementary Tables S3 and S4). FL Follicular lymphoma, DLBCL Diffuse large B-cell lymphoma, ABC activated B-cell-like, GCB germinal center B-cell-like, TCC tumor cell content, WGS whole genome sequencing, r rearranged, n. a. not applicable.

While DNA methylation (DNAme) has emerged as biomarker for tumor classification, e.g. in brain tumors or sarcomas [9, 10], its use for the subtyping of gcBC lymphomas, including FL and DLBCL, is still lagging behind. Therefore, we performed BeadChip array-based DNAme analysis on DNA extracted from 177 molecularly well-characterized cases of gcBC lymphomas included in the ICGC MMML-Seq consortium. These comprised cryo-preserved tumor cell-rich tissues of 85 FL, 75 DLBCL, and 17 FL-DLBCL (Supplementary Table S1), as presented by Hübschmann and colleagues [8]. In standard clustering and dimension-reduction analyses of the 10,000 most variable CpGs of this dataset, FL and DLBCL appear as a continuum or cloud of cases, mostly organized by the amount of DNAme of the investigated CpGs (Supplementary Fig. S1). To nevertheless identify potential subgroups of these lymphomas, we applied the Phenotype-Genotype Many-to-Many Relations Analysis (PGMRA) algorithm to the data [11]. The PGMRA algorithm is an unsupervised machine learning method that employs a fuzzy non-negative matrix factorization method to identify significant biclusters of features (e.g. CpG loci) and cases. For this approach, we first selected the 10,000 most variable CpGs based on standard deviation and applied k-means clustering to organize them into 1000 clusters. By selecting representative CpGs from each cluster, we reduced the CpG set to 1938 CpGs. These CpGs were then analyzed using PGMRA to identify significant biclusters, i.e. clusters of both CpGs and lymphoma samples (Supplementary methods). PGMRA identified 300 significant CpGs across 119 significant samples. We applied k-means clustering to organize the 300 CpGs and 177 samples (Fig. 1A). This analysis revealed four CpG modules (M1: 68 CpGs, M2: 50 CpGs, M3: 94 CpGs, M4: 88 CpGs, Supplementary Table S2), which organized the lymphomas into seven distinct methylation patterns (MP1-MP7, Supplementary Fig. S2).

By correlating the methylation patterns (MPs) with the recently published epidemiologic, histopathologic, transcriptomic and genetic aberration characteristics of these lymphomas [8], we unraveled strong, though non-perfect, association with the histopathologic diagnoses, with two MPs mainly containing FL, four MPs mostly containing DLBCL, leaving one intermediate MP (Fig. 1B).

In particular, MP1 and MP2 are predominantly composed of FL cases (MP1: 37/41 [90%]; MP2: 24/36 [67%]), with MP2 showing a higher age at diagnosis compared to MP1 (median [range] age: MP2: 70 [44–78] years vs. MP1: 60 [33–80] years; *p* = 0.047) (Fig. 1C). Additionally, MP2 displays a higher proliferation history, calculated using the mitotic clock epiCMIT [12], compared to MP1 (median [range]: MP2: 0.7 [0.6–0.9] vs. MP1: 0.6 [0.4–0.7]; *p* < 0.001) and a higher mutational load of single nucleotide variants (median [range]: MP2: 8439 [4512–31,343] vs. MP1: 4658 [1335–12,417]; *p* < 0.001) (Supplementary Fig. S3). Both MP1 and MP2 exhibit a high frequency of cases with *BCL2* rearrangement (MP1: 36/41 [88%]; MP2: 36/36 [100%]) and GCB subtype (MP1: 32/41 [78%]; MP2: 28/36 [78%]), in line with the enrichment of FL. Intriguingly, while our DNAme analysis revealed distinct patterns related with clinical characteristics for MP1 and MP2, these differences were not reflected in the RNA expression profiles. MP3 comprises a mixture of FL (13/22 [59%]), DLBCL (8/22 [36%]) and FL-DLBCL (1/22 [5%]) cases. It is characterized by fewer cases with *BCL2* rearrangement (11/22 [50%]) compared to MP1 and MP2 (*p* < 0.001), while similarly showing a predominance of the GCB subtype (17/22 [77%]).

The remaining MPs, i.e. MP4-7, consist in the majority of DLBCL cases (MP4: 20/28 [71%]; MP5: 17/26 [65%]; MP6: 10/11 [91%], MP7: 11/13 [85%]). Among these, MP4 and MP5 mainly contain cases of GCB subtype (MP4: 21/28 [82%]; MP5: 16/26 [62%]), while MP6 and MP7 are associated with the ABC subtype (MP6: 10/11 [91%]; MP7: 7/13 [54%]) (Supplementary Table S3). Using the mutational clusters identified by Hübschmann et al. for the whole set of cases as well as for the DLBCL subset [8], we found that MP4 exhibits the highest proportion of cases belonging to the *PIM1*-like mutational cluster derived from clustering of the entire dataset (OR = 6.1, *p* = 0.001). Furthermore, MP6 shows an enrichment of cases of the mutational cluster containing *MYD88* and/or *CD79B* mutation as hallmark (entire dataset: OR = 26.0, *p* < 0.001), thus reflecting the so-called C5/MCD cluster [3, 4]. This is in line with the ABC subtype enrichment (OR = 78.6, *p* < 0.001) and absence of *BCL2* rearrangements (0/11 [0%]) in MP6. While nodal involvement was higher in MP1 (*p* = 0.014) likely due to the enrichment of FL cases, we observed no significant differences between the MPs in clinical parameters like stage or International Prognostic Index, given the low power of the analyses relying on small sample sizes (Supplementary Fig. S4).

To elucidate whether the DNAme clustering might be driven by tumor or bystander cells, we evaluated the tumor cell content (TCC) using whole genome sequencing data and several DNAme-based purity parameters (Supplementary Fig. S5). The lowest median TCC was detected in MP3 (30%) and MP4 (40%). Remarkably, MP4 and MP5 exhibit similarities in the DNAme for the CpG modules M1–M3, besides these CpGs are less methylated in MP4 as compared to MP5 cases (median [range] beta-value for M1-3: MP4: 0.41 [0.09–0.51] vs. MP5: 0.61 [0.50–0.85]; *p* < 0.001) (Supplementary Fig. S6). This lower DNAme, coupled with a significantly lower proliferation history (*p* < 0.001), B-cell presence (*p* < 0.001), and TCC (*p* < 0.001) as compared to MP5, correlates with a higher predicted proportion of bystander cells within MP4 cases (CD4^+^ T-cells *p* < 0.001).

**Fig. 2:**
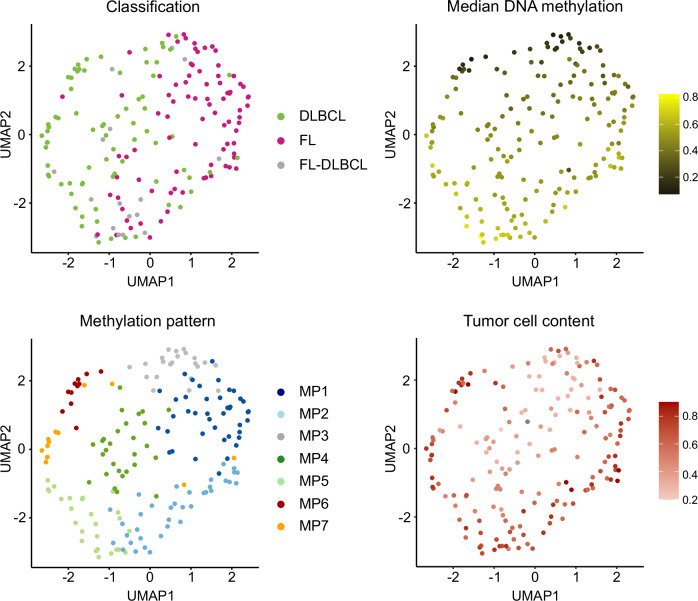
UMAP analysis of the 300 CpGs in germinal center-derived B-cell lymphomas. UMAP analysis based on 300 CpGs identified by PGMRA, using Manhattan distance and 15 neighbors. The UMAP plots are colored according to various features: lymphoma classification, median DNA methylation levels, methylation patterns (MPs) and tumor cell content based on whole genome sequencing. FL Follicular lymphoma, DLBCL Diffuse large B-cell lymphoma.

We next conducted a Uniform Manifold Approximation and Projection (UMAP) analysis based on the 300 CpGs (Fig. 2, Supplementary Fig. S7). Notably, UMAP1 (x-axis) stratifies samples according to their histopathologic diagnoses while UMAP2 (y-axis) correlates with the median DNAme levels. Furthermore, although a continuum is still present within this sample distribution, discernible clustering tendencies emerge, which expectedly align well with the seven MPs previously identified through k-means clustering.

To elucidate how the 300 CpGs perform on other common mature B-cell lymphomas derived from gcBCs, we included into the UMAP additional array-based DNAme data from cryo-preserved tissues of 31 sporadic EBV-negative Burkitt lymphomas (BL), 7 high-grade B-cell lymphomas with 11q aberration (HGBCL-11q) and 7 nodal marginal zone lymphomas (nMZL) from the MMML cohorts (Supplementary Fig. S8) [13–15]. BL segregated clearly apart as separate cluster from DLBCL, FL and also HGBCL-11q, suggesting that the selected CpGs might also be able to differentiate BL and HGBCL-11q. Notably, nMZL cases showed heterogeneous methylation profiles and, thus, clustered into the areas of several MPs, probably due to lower TCC or diverse biological backgrounds. To validate our findings, we analyzed publicly available datasets of DLBCLs (*n* = 69) and primary central nervous system lymphomas (PCNSLs; Carlund et al.: *n* = 8, Vogt et al.: *n* = 26) [16, 17]. DLBCLs were mainly distributed across MP4-6 similarly to the DLBCL of the ICGC MMML-Seq cohort. In contrast, the PCNSLs known to mostly belong to the MCD/C5 group, predominantly clustered with cases in MP7 (33/34 [97%]) enriched for DLBCL of the MYD88 subgroup within the ICGC MMML-Seq cohort (Supplementary Fig. S9). By displaying the CpG modules to non-malignant (pre-)B-cell subpopulations, we found that the DNAme levels of these 300 CpGs are remarkably uniform, with low DNAme in M1-3 (median [range] beta-value: M1: 0.05 [0.03–0.22]; M2: 0.05 [0.03–0.18]; M3: 0.06 [0.03–0.29]) and mostly high DNAme in M4 (median [range] beta-value: 0.86 [0.69–0.91]) (Supplementary Fig. S10).

Finally, we aimed at investigating the 300 CpGs in more detail though they were not selected for biologic function (Supplementary Fig. S11). Modules M1-3, showing varying DNAme levels across the seven MPs, are significantly enriched within CpG islands (M1–3: *p* < 0.001), in poised promoter regions defined in gcBCs (M2-3: *p* < 0.001), and in bivalent transcription start sites defined in a human embryonic stem cell line (M2-3: *p* < 0.001). They belong predominantly to module 20 (M1: 75%; M2: 100%, M3: 83%) of the dynamically methylated CpGs during B-cell development described by Kulis et al. [18] (Supplementary Table S5). The increase in DNAme levels particularly in M3 in part correlates with the number of cell cycles the tumor cells had experienced within the germinal center (Supplementary Fig. S12). Conversely, CpGs in M4 are located in enhancer, transcription, and heterochromatic regions, not linked to CpG islands. Despite their predominately high methylation (median [range] beta-value: 0.66 [0.24–0.88]) in M4 they contribute to the MP structure and, thus, potentially hold significant value as biomarkers for diagnostic and prognostic applications in lymphoid malignancies.

DNAme-based classification of several solid tumor types has entered clinical practice and DNAme studies of predominately leukemic haematologic neoplasms including B- and T-cell leukemias have shown clear subgroups based on the lineages and maturation stages of the tumor cells [18]. Despite this progress, DNAme-based grouping of the most common mature B-cell lymphomas, i.e. FL and DLBCL, has been challenging and mostly revealed an amorphic crowd of cases with a continuum of DNAme levels (Supplementary Fig. S1). Here, the use of unsupervised fuzzy non-negative matrix factorization methods, identified 300 CpGs in DNA from cryo-preserved FL and DLBCL that categorize these lymphomas into subgroups. These subgroups correlate with known mutational groups but also reflect biological features like age at diagnosis and proliferation history. Thus, they provide subgrouping information orthogonal to current systems using morphology, transcriptomics, or genetic alterations. Though not designed for this aim, we show that the selected CpG modules and DNAme profiles also have the potential to differentiate other gcBC lymphomas, like BL, as well as non-malignant (pre-)B-cell populations. Both the underlying PGMRA approach as well as the presented set of CpGs, if validated in independent cohorts, might in the future contribute to the application of DNAme biomarkers in common lymphomas similar to other tumor entities.

## Supplementary information

Supplementary Methods

Supplementary Figures

Supplementary Tables S1-S5

## Data Availability

DNA methylome data produced in this study are available at GEO under accession number GSE276853.
