# Supplementary material for: Subgrouping germinal center-derived B-cell lymphomas based on machine learning-deduced DNA methylation modules
Source: Leukemia. 2025 Mar 10;39(4):967–71. doi: 10.1038/s41375-025-02533-6 (PMC11976257; doi:10.1038/s41375-025-02533-6)
Supplement: Supplementary file 1 — Supplementary Methods [file 41375_2025_2533_MOESM1_ESM.docx]

# Supplementary Information

## Full list of members of the ICGC MMML-Seq

*Coordination (C1):* Reiner Siebert^1,2^, Susanne Wagner^2^, Andrea Haake^2^, Julia Richter^2,3^, Gesine Richter^2^

*Data Center (C2):* Roland Eils^4,5^, Chris Lawerenz^4^, Jürgen Eils^4^, Jules Kerssemakers^4^, Christina Jaeger-Schmidt^4^, Ingrid Scholz^4^

*Clinical Centers (WP1):* Anke K. Bergmann^2, 6^*,* Christoph Borst^7^, Birgit Burkhardt^8,9^, Alexander Claviez^6^, Martin Dreyling^10^, Sonja Eberth^11^, Hermann Einsele^12^, Norbert Frickhofen^13^, Siegfried Haas^7^, Martin-Leo Hansmann^14^, Dennis Karsch^15^, Michael Kneba^15^, Jasmin Lisfeld^9^, Luisa Mantovani-Löffler^16^, Marius Rohde^9^, German Ott^17^, Christina Stadler^11^, Peter Staib^18^, Stephan Stilgenbauer^19^, Lorenz Trümper^11^, Thorsten Zenz^20^

*Normal Cells (WPN):* Martin-Leo Hansmann^14^, Dieter Kube^11^, Ralf Küppers^21^, Marc Weniger^21^

*Pathology and Analyte Preparation (WP2-3):* Michael Hummel^22^, Wolfram Klapper^3^, Ulrike Kostezka^23^, Dido Lenze^22^, Peter Möller^24^, Andreas Rosenwald^25^, German Ott^17^, Monika Szczepanowski^3^

*Sequencing and genomics (WP4-7):* Ole Ammerpohl^1,2^, Sietse M. Aukema^2,3^, Vera Binder^26^, Arndt Borkhardt^26^, Andrea Haake^2^, Jessica I. Hoell^26^; Ellen Leich^25^, Peter Lichter^27^, Cristina López^1,2^, Inga Nagel^2^, Jordan Pischimariov^25^, Bernhard Radlwimmer^27^, Julia Richter^2,3^, Philip Rosenstiel^28^, Andreas Rosenwald^25^, Markus Schilhabel^28^, Stefan Schreiber^29^, Inga Vater^2^, Rabea Wagener^1,2^, Reiner Siebert^1,2^

*Bioinformatics (WP8-9):* Stephan H. Bernhart^30-32,^ Hans Binder^30,31^, Benedikt Brors^33^, Gero Doose^30-32^, Roland Eils^4,5^, Steve Hoffmann^30-32^, Lydia Hopp^30^, Daniel Hübschmann^4,5,34^, Kortine Kleinheinz^4,5^, Helene Kretzmer^30-32^, Markus Kreuz^35^, Jan Korbel^36^, David Langenberger^30-32^, Markus Loeffler^35^, Maciej Rosolowski^35^, Matthias Schlesner^4,37^, Peter F. Stadler^30-32,38-40^, Stephanie Sungalee^36^

^1^Institute of Human Genetics, University of Ulm and University Hospital of Ulm, Ulm, Germany

^2^Institute of Human Genetics, Christian-Albrechts-University, Kiel, Germany;

^3^Hematopathology Section, Institute of Pathology, Christian-Albrechts-University, Kiel, Germany;

^4^Division of Theoretical Bioinformatics (B080), German Cancer Research Center (DKFZ), Heidelberg, Germany;

^5^Department for Bioinformatics and Functional Genomics, Institute of Pharmacy and Molecular Biotechnology and Bioquant, University of Heidelberg, Heidelberg, Germany;

^6^Department of Pediatrics, University Hospital Schleswig-Holstein, Campus Kiel, Kiel, Germany;

^7^Department of Internal Medicine/Hematology, Friedrich-Ebert-Hospital, Neumünster;

^8^University Hospital Muenster - Pediatric Hematology and Oncology, Muenster Germany;

^9^University Hospital Giessen, Pediatric Hematology and Oncology, Giessen, Germany;

^10^Department of Medicine III - Campus Grosshadern, University Hospital Munich, Munich, Germany;

^11^Department of Hematology and Oncology, Georg-August-University of Göttingen, Göttingen, Germany;

^12^University Hospital Würzburg, Department of Medicine and Poliklinik II, University of Würzburg, Würzburg;

^13^Department of Medicine III, Hematology and Oncology, Dr. Horst-Schmidt-Kliniken of Wiesbaden, Wiesbaden;

^14^Senckenberg Institute of Pathology, University of Frankfurt Medical School, Frankfurt am Main, Germany

^15^Department of Internal Medicine II: Hematology and Oncology, University Medical Centre, Campus Kiel, Kiel;

^16^Hospital of Internal Medicine II, Hematology and Oncology, St-Georg Hospital Leipzig, Leipzig, Germany;

^17^Department of Clinical Pathology, Robert-Bosch Krankenhaus, and Dr. Margarete Fischer-Bosch Institute of Clinical Pharmacology, Stuttgart, Germany;

^18^Clinic for Hematology and Oncology, St.-Antonius-Hospital, Eschweiler;

^19^Department for Internal Medicine III, University of Ulm and University Hospital of Ulm, Ulm, Germany

^20^National Centre for Tumor Disease, Heidelberg, Germany;

^21^Institute of Cell Biology (Cancer Research), University of Duisburg-Essen, Medical School, Essen, Germany;

^22^CharitéCenter for Biomedicine (CC4), Charité – University Medicine Berlin, Berlin, Germany;

^23^Comprehensive Cancer Center Ulm (CCCU), University Hospital Ulm, Ulm, Germany;

^24^Institute of Pathology, University of Ulm and University Hospital of Ulm, Ulm;

^25^Institute of Pathology, University of Wurzburg, Germany;

^26^Department of Pediatric Oncology, Hematology and Clinical Immunology, Heinrich-Heine-University, Düsseldorf, Germany;

^27^German Cancer Research Center (DKFZ), Division of Molecular Genetics, Heidelberg, 69120, Germany;

^28^Institute of Clinical Molecular Biology, Christian-Albrechts-University, Kiel, Germany;

^29^Department of General Internal Medicine, University Kiel, Kiel, Germany;

^30^Interdisciplinary Center for Bioinformatics, University of Leipzig, Leipzig, Germany;

^31^Bioinformatics Group, Department of Computer, University of Leipzig, Leipzig, Germany; ^32^Transcriptome Bioinformatics, LIFE Research Center for Civilization Diseases, University of Leipzig, Leipzig, Germany;

^33^Division of Applied Bioinformatics (G200), German Cancer Research Center (DKFZ), Heidelberg, Germany

^34^Department of Pediatric Immunology, Hematology and Oncology, University Hospital, Heidelberg, Germany

^35^Institute for Medical Informatics Statistics and Epidemiology, University of Leipzig, Leipzig, Germany;

^36^EMBL Heidelberg, Genome Biology, Heidelberg, Germany;

^37^Bioinformatics and Omics Data Analytics (B240), German Cancer Research Center (DKFZ), Heidelberg, Germany;

^38^RNomics Group, Fraunhofer Institute for Cell Therapy and Immunology IZI, Leipzig, Germany

^39^Santa Fe Institute, Santa Fe, New Mexico, United States of America

^40^Max-Planck-Institute for Mathematics in Sciences, Leipzig, Germany.

# Supplementary Methods

## Study cohort

We investigated 177 germinal center-derived B-cell (gcBC) lymphomas, including 85 follicular lymphomas (FL) (median age at diagnosis: 62 years [33-80]; female: 59%), 75 diffuse large B-cell lymphomas (DLBCL) (median age at diagnosis: 62 years [33-89]; female: 48%), and 17 follicular lymphomas with diffuse large B-cell lymphoma (FL-DLBCL) (median age at diagnosis: 72 years [43-79]; females: 65%), from the ICGC MMML-Seq network. The detailed histopathologic, transcriptional and genomic characterization of these cases, which by the inclusion criteria were enriched for samples with high tumor cell content, has been published by Hübschmann et al. (1). Main features of the cases are summarized in Supplementary Table S1, which is an adapted and amended version of Supplementary Table S1A in Hübschmann et al. For comparison, we included 31 sporadic EBV-negative Burkitt lymphomas (median age at diagnosis: 10 years [2-40]; females: 19%) and 7 high-grade B-cell lymphoma with 11q aberration (median age at diagnosis: 17 years [8-76]; females: 29%) from the ICGC MMML-Seq and MMML projects (2–4) and 7 nodal marginal zone lymphoma (median age at diagnosis: 72 years [60-82]; female: 43%) from the ICGC MMML-Seq. The MMML and ICGC MMML-Seq studies have been approved by the Institutional Review Board of the Medical Faculties of the University of Kiel (403/05 and A150/10) and Ulm (349/11 for ICGC MMML-Seq). Informed consent from the patients or their legal guardians was obtained in accordance with the respective regulations of the institutional review boards. Furthermore, we included publically available data from 69 DLBCL cases and 34 primary central nervous system lymphoma (5,6).

In addition, we mined previously published data from non-malignant (pre-)B-cell subpopulations, including hematopoietic stem cells, pre B-cells, immature B-cells, naïve B-cells, tonsillar naïve B-cells, germinal center founder cells, germinal center B-cells, early non class-switched B-cells, non class-switched memory B-cells, class-switched memory B-cells, splenic marginal zone B-cells, tonsillar plasma cells and bone marrow plasma B-cells (7–10).

## DNA methylation BeadChip arrays

For DNA methylation analysis, 500-1000 ng of DNA from cryo-preserved tissue was bisulfite converted using the EZ DNA Methylation Kit (Zymo Research, Irvine, CA, USA) according to the manufacturer's instructions and hybridized on the Infinium HumanMethylation450 BeadChip array (450k) from Illumina (Illumina, San Diego, CA, USA). The raw idat files were pre-processed in R using minfi package (version 1.48.0) with the preprocessIllumina function against intrinsic controls, and with no background correction. Afterwards, CpGs with a p-value < 0.01, CpGs on gonosomes, and rs loci were excluded. Finally, 473 864 CpGs underwent further investigation.

From the 181 cases listed in Hübschmann et al. (1), we considered only FL, DLBCL and FL-DLBCL cases, resulting in a subset of 178 cases. Of the 178 samples a total of 237 arrays entered the analyses. One case had to be excluded as it did not pass the quality measures, while in cases with duplicate arrays those with the best technical performance were included. Thus, a total of 177 arrays representing a total of 177 cases entered the final analysis.

DNA methylation BeadChip array analysis of the 31 sporadic EBV-negative “solid” BL, the 7 HGBCL-11q aberration and 7 nMZL from the MMML networks were processed using the ICGC MMML-Seq pipeline. Of the 45 cases, 80 arrays were analysed. Again, for cases with replicated arrays, those with the best technical performance were included.

## Feature selection strategy using PGMRA

As input for this approach, the 10 000 most variable CpGs were selected based on the standard deviation. In a first step, we applied a dimensionality reduction technique to the dataset that involved the use of k-means clustering to organize the CpGs into 1 000 clusters. In a second step, the clusters were sorted according to the number of CpGs they included. From clusters containing 1 to 3 CpGs, we selected all CpGs; from clusters containing between 4 and 10 CpGs, we selected the antipodal CpGs, those CpGs in a cluster that have the maximum distance between them, and the centroid CpGs. For the clusters with more than 10 CpGs, we selected only the centroid. This process allowed us to effectively reduce the number of features to 1 938 CpGs while maintaining the most variable CpGs in the original cohort.

The selected 1 938 CpGs were further analyzed using PGMRA, an unsupervised machine learning method, that selects optimal biclusters using non-dominant niche optimization techniques often used in evolutionary computation (11). PGMRA uses fuzzy non-negative matrix factorization to identify significant biclusters, which are groups of samples that present similar value profiles for a group of CpGs (12). Each bicluster was tested against other biclusters using F-statistics on features (CpGs) within the bicluster only (p-value <0.005). The CpGs from the selected PGMRA optimal biclusters were extracted and used to analyze the cohort.

## Chromatin Mapping

The chromatin states from gcBCs described by Hübschmann et al. were used (1). Chromatin states for the human stem cell line HUES64 were downloaded from the Roadmap Epigenomics Project (https://egg2.wustl.edu/roadmap/web_portal/index.html).

## Statistical analysis

All statistical analyses were performed in R (version 4.3.0). The proliferative history was assessed using the epigenetically-determined cumulative mitoses (epiCMIT) in R (13). To calculate various purity scores based on DNA methylation data, we used the R packages, InfiniumPurify (version 2.0) (14), RFPurify (version 0.1.2) (15), and Flow.Sorted.Blood.450k (version 1.38.0) (16). The optimal number of clusters was calculated using the package cluster (version 2.1.6) (Supplementary Figure S2) (17). The Wilcoxon rank sum test was used for pairwise comparisons between independent groups described by continuous variables. For categorical variables Fisher’s exact test was used to calculate odds ratios and p-values. As background set, either the 300 CpGs or all samples were used. The Bonferroni method was used to adjust p-values for multiple comparison. A difference was considered to be statistically significant when an adjusted p-value was less than 0.05 (Supplementary Tables S3-5).

## References

1. Hübschmann D, Kleinheinz K, Wagener R, Bernhart SH, López C, Toprak UH, et al. Mutational mechanisms shaping the coding and noncoding genome of germinal center derived B-cell lymphomas. Leukemia. 2021 Jul;35(7):2002–16.

2. López C, Kleinheinz K, Aukema SM, Rohde M, Bernhart SH, Hübschmann D, et al. Genomic and transcriptomic changes complement each other in the pathogenesis of sporadic Burkitt lymphoma. Nat Commun. 2019 Mar 29;10(1):1459.

3. Kretzmer H, Bernhart SH, Wang W, Haake A, Weniger MA, Bergmann AK, et al. DNA methylome analysis in Burkitt and follicular lymphomas identifies differentially methylated regions linked to somatic mutation and transcriptional control. Nat Genet. 2015 Nov;47(11):1316–25.

4. Loeffler-Wirth H, Kreuz M, Schmidt M, Ott G, Siebert R, Binder H. Classifying Germinal Center Derived Lymphomas—Navigate a Complex Transcriptional Landscape. Cancers. 2022 Jul 14;14(14):3434.

5. Carlund O, Thörn E, Osterman P, Fors M, Dernstedt A, Forsell MNE, et al. Semimethylation is a feature of diffuse large B-cell lymphoma, and subgroups with poor prognosis are characterized by global hypomethylation and short telomere length. Clin Epigenet. 2024 May 21;16(1):68.

6. Vogt J, Wagener R, Montesinos-Rongen M, Ammerpohl O, Paulus W, Deckert M, et al. Array-based profiling of the lymphoma cell DNA methylome does not unequivocally distinguish primary lymphomas of the central nervous system from non-CNS diffuse large B-cell lymphomas. Genes Chromosomes Cancer. 2019 Jan;58(1):66–9.

7. Lee ST, Xiao Y, Muench MO, Xiao J, Fomin ME, Wiencke JK, et al. A global DNA methylation and gene expression analysis of early human B-cell development reveals a demethylation signature and transcription factor network. Nucleic Acids Research. 2012 Dec;40(22):11339–51.

8. Kulis M, Merkel A, Heath S, Queirós AC, Schuyler RP, Castellano G, et al. Whole-genome fingerprint of the DNA methylome during human B cell differentiation. Nat Genet. 2015 Jul;47(7):746–56.

9. Oakes CC, Seifert M, Assenov Y, Gu L, Przekopowitz M, Ruppert AS, et al. DNA methylation dynamics during B cell maturation underlie a continuum of disease phenotypes in chronic lymphocytic leukemia. Nat Genet. 2016 Mar;48(3):253–64.

10. ICGC MMML-Seq project, BLUEPRINT project, Kretzmer H, Bernhart SH, Wang W, Haake A, et al. DNA methylome analysis in Burkitt and follicular lymphomas identifies differentially methylated regions linked to somatic mutation and transcriptional control. Nat Genet. 2015 Nov;47(11):1316–25.

11. Zwir I, Arnedo J, Mesa A, Del Val C, De Erausquin GA, Cloninger CR. Temperament & Character account for brain functional connectivity at rest: A diathesis-stress model of functional dysregulation in psychosis. Mol Psychiatry. 2023 Jun;28(6):2238–53.

12. Arnedo J, Del Val C, De Erausquin GA, Romero-Zaliz R, Svrakic D, Cloninger CR, et al. PGMRA: a web server for (phenotype x genotype) many-to-many relation analysis in GWAS. Nucleic Acids Research. 2013 Jul 1;41(W1):W142–9.

13. Duran-Ferrer M, Clot G, Nadeu F, Beekman R, Baumann T, Nordlund J, et al. The proliferative history shapes the DNA methylome of B-cell tumors and predicts clinical outcome. Nat Cancer. 2020 Nov 2;1(11):1066–81.

14. Qin Y, Feng H, Chen M, Wu H, Zheng X. InfiniumPurify: An R package for estimating and accounting for tumor purity in cancer methylation research. Genes & Diseases. 2018 Mar;5(1):43–5.

15. Johann PD, Jäger N, Pfister SM, Sill M. RF_Purify: a novel tool for comprehensive analysis of tumor-purity in methylation array data based on random forest regression. BMC Bioinformatics. 2019 Aug 16;20(1):428.

16. Jaffe AE. FlowSorted.Blood.450k [Internet]. Bioconductor; 2017 [cited 2024 Aug 2]. Available from: https://bioconductor.org/packages/FlowSorted.Blood.450k

17. Maechler M, Rousseeuw P, Struyf A, Hubert M. cluster: ‘Finding Groups in Data’: Cluster Analysis Extended Rousseeuw et al. [Internet]. 1999 [cited 2024 Aug 5]. p. 2.1.6. Available from: https://CRAN.R-project.org/package=cluster
