# Supplementary material for: Subgrouping germinal center-derived B-cell lymphomas based on machine learning-deduced DNA methylation modules
Source: Leukemia. 2025 Mar 10;39(4):967–71. doi: 10.1038/s41375-025-02533-6 (PMC11976257; doi:10.1038/s41375-025-02533-6)
Supplement: Supplementary file 2 — Supplementary Figures [file 41375_2025_2533_MOESM2_ESM.pdf]

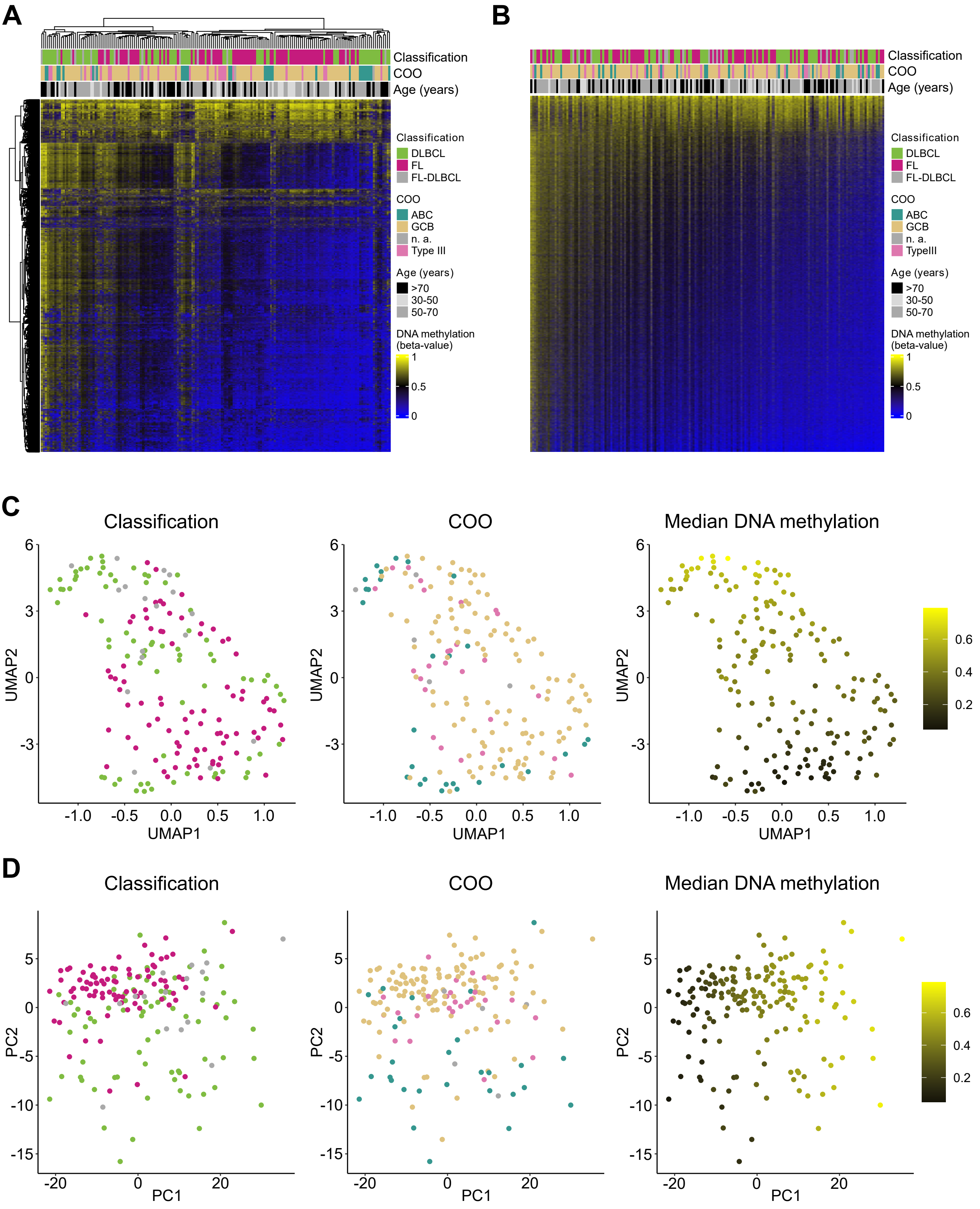

Supplementary Figure S1: DNA methylation profiling of germinal center-derived B-cell lymphomas using the 10 000 most variable CpGs. A, B: Heatmaps displaying the 10 000 most variable CpGs of 177 germinal center-dervied B-cell lymphomas (FL, DLBCL, FL-DLBCL)**.** Rows represent individual CpGs, and columns represent samples. CpG sites and samples are organized either by hierarchical clustering (A) or by median DNA methylation (B). **C, D:** UMAP (Manhattan distance, 15 neighbors, C) and principal component (D) analysis based on the 10 000 most variable CpGs. Plots are colored according different annotations: Classification, COO and median DNA methylation. Legend description as illustrated in A and B. FL: Follicular lymphoma; DLBCL: Diffuse large B-cell lymphoma; COO: Cell-of-origin.

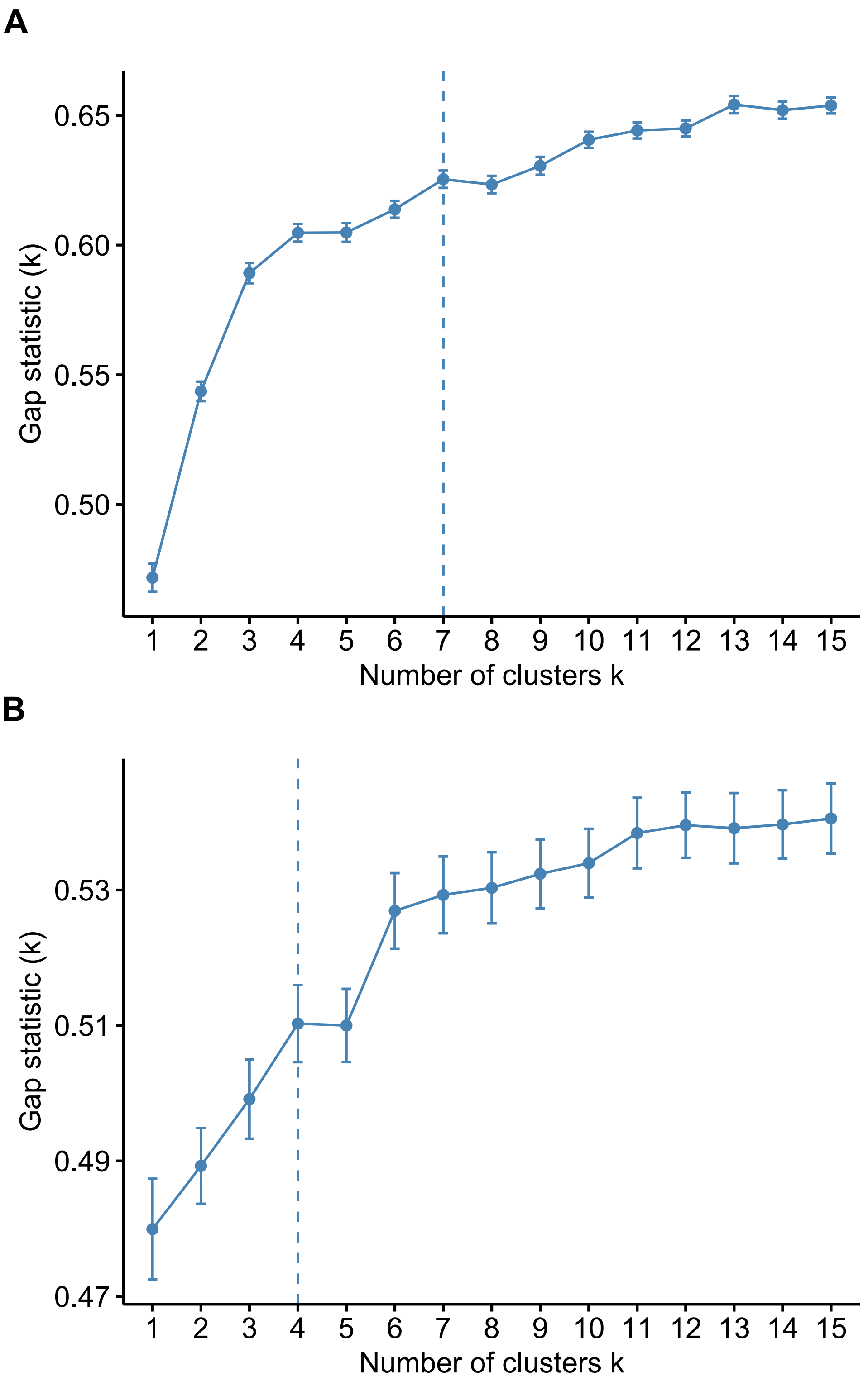

Supplementary Figure S2: Calculation of the optimal number of k-means clusters. Calculation of the optimal number of k-means clusters was performed with the R package cluster using the 300 CpGs selected by PGMRA. Dashed line represents suggested optimal number of k-means clusters. **A:** Samples, Methylation patterns. **B:** CpGs, CpG modules.

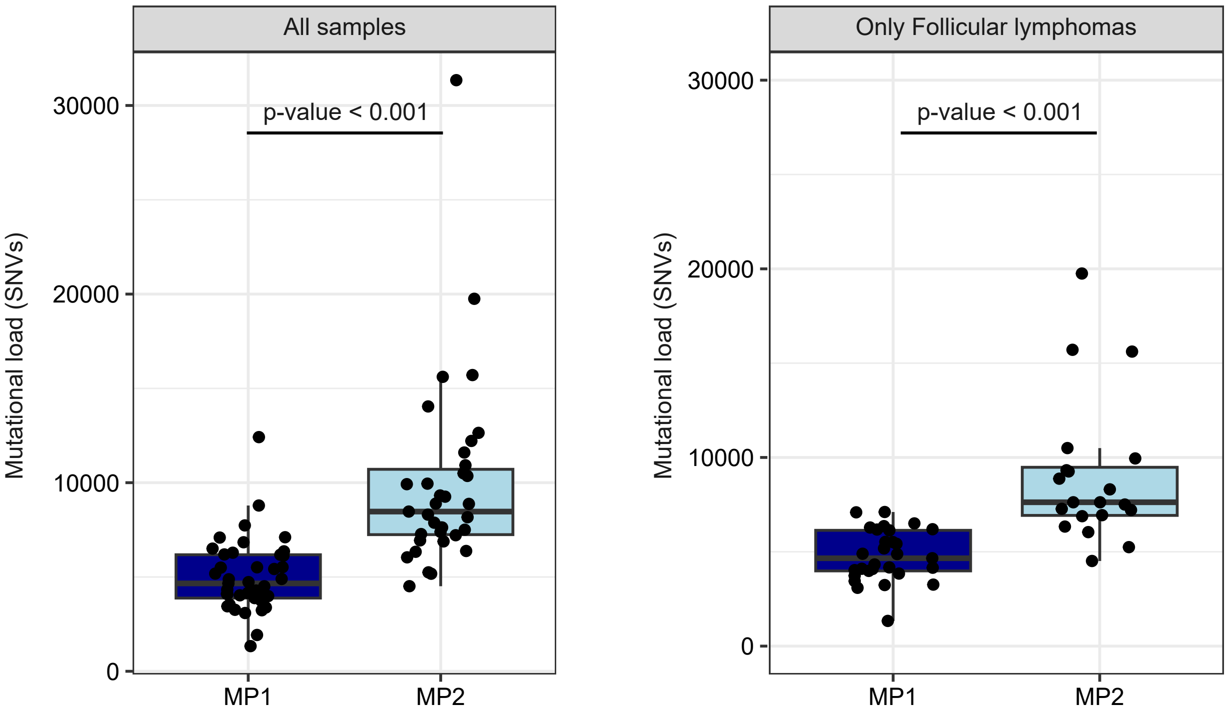

Supplementary Figure S3: Mutational load across methylation patterns MP1 versus MP2. Wilcoxon rank sum test was used for pairwise comparison. SNVs: single nucelotide variants.

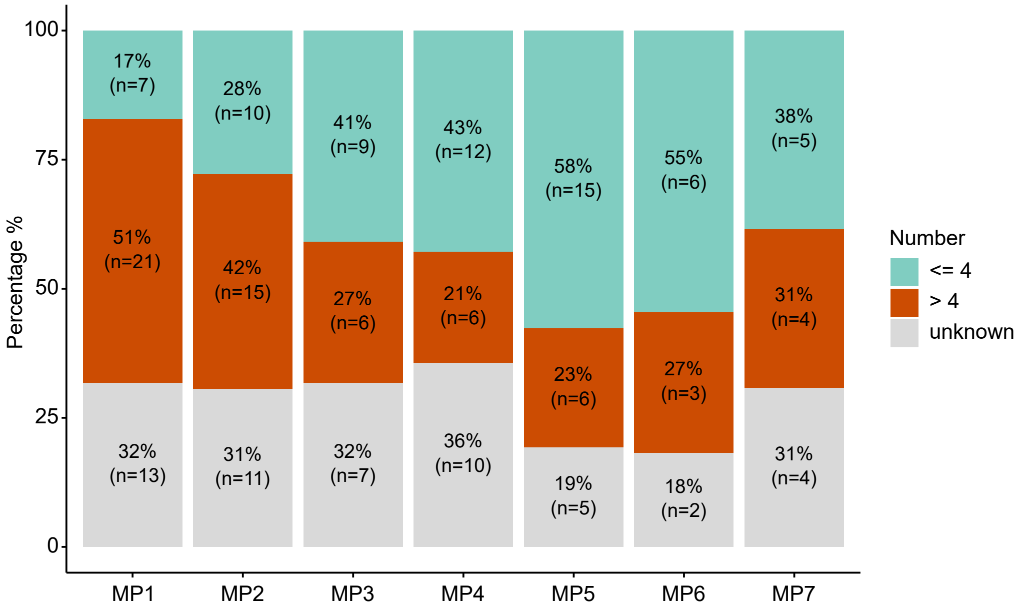

Supplementary Figure S4: Distribution of nodal involvement across methylation patterns (MPs). Barplot depicting the proportion of cases with nodal involvement for each of the seven methylation patterns (MP1-7). Nodal involvement is categorized as either less than or equal to 4 regions or more than 4 regions.

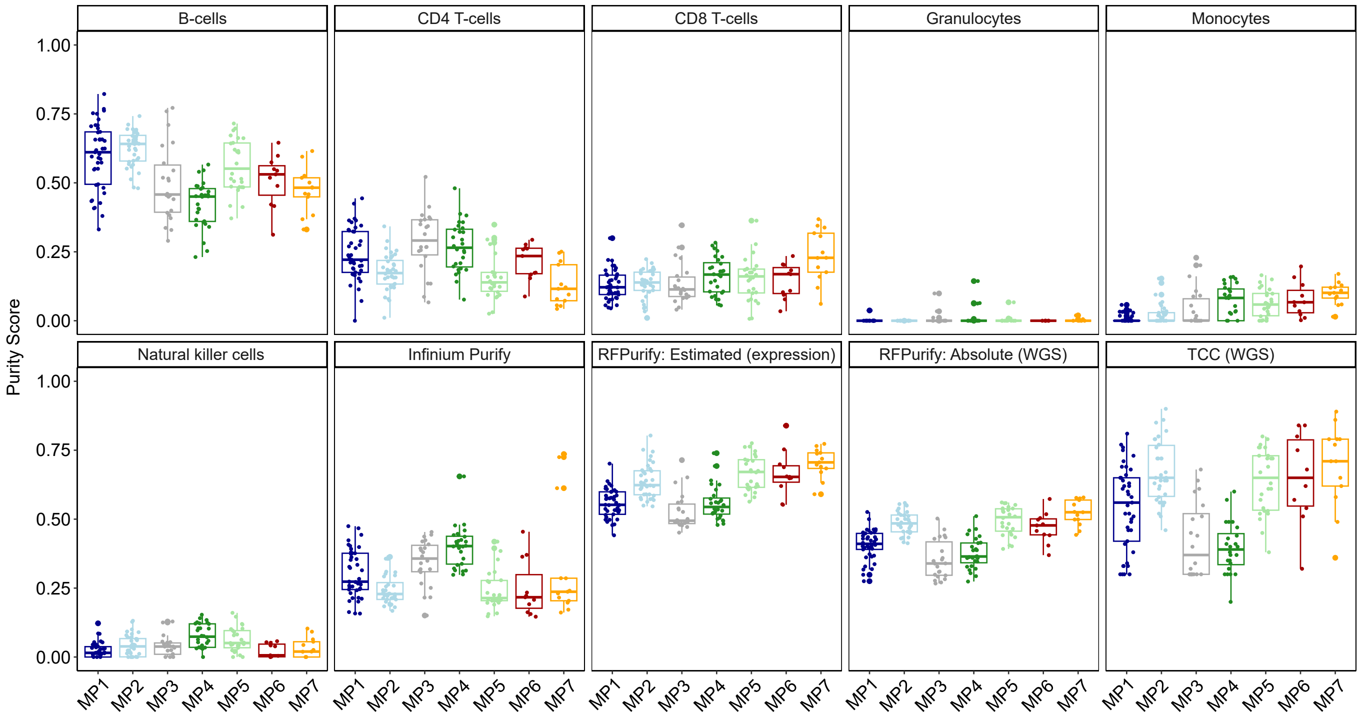

Supplementary Figure S5: DNA methylation-based calculations of cellular composition and purity scores across the seven methylation patterns. The boxplots display various purity scores, cellular composition and tumor cell content (TCC) calculated using whole genome sequencing data (WGS). Statistical analysis is summarized in Supplementary Table S4.

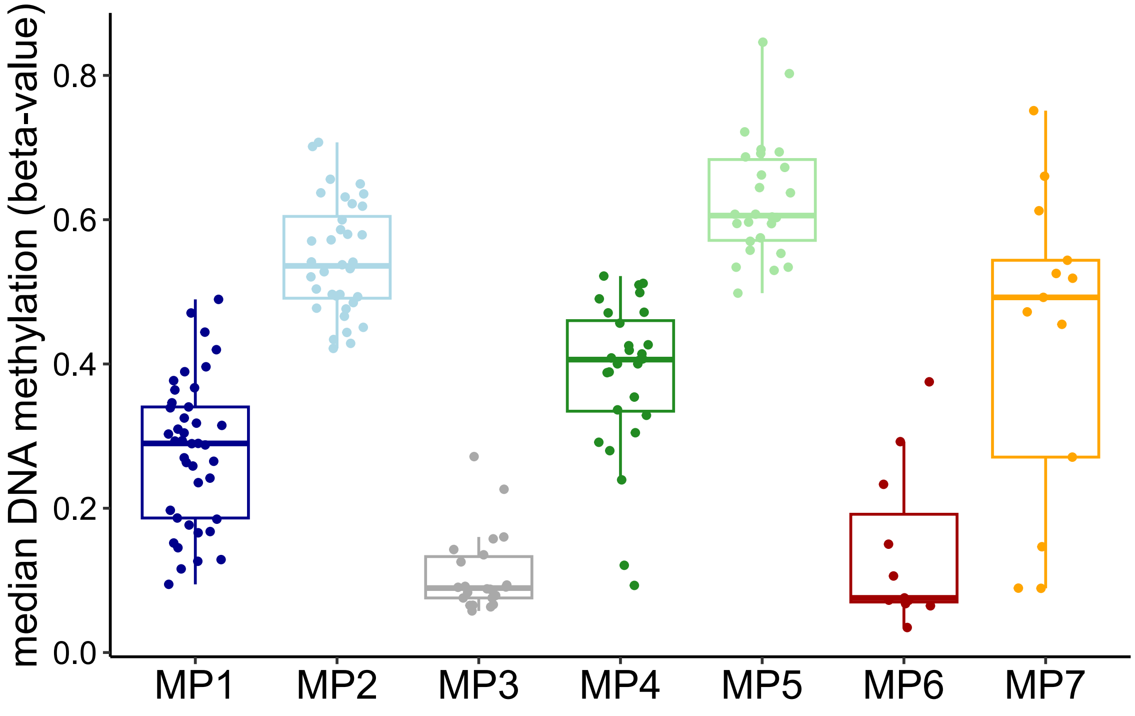

Supplementary Figure S6: Median DNA methylation levels of the CpG modules M1-3 across the seven methylation patterns (MPs). Statistical comparisons are summarized in Supplementary Table S4.

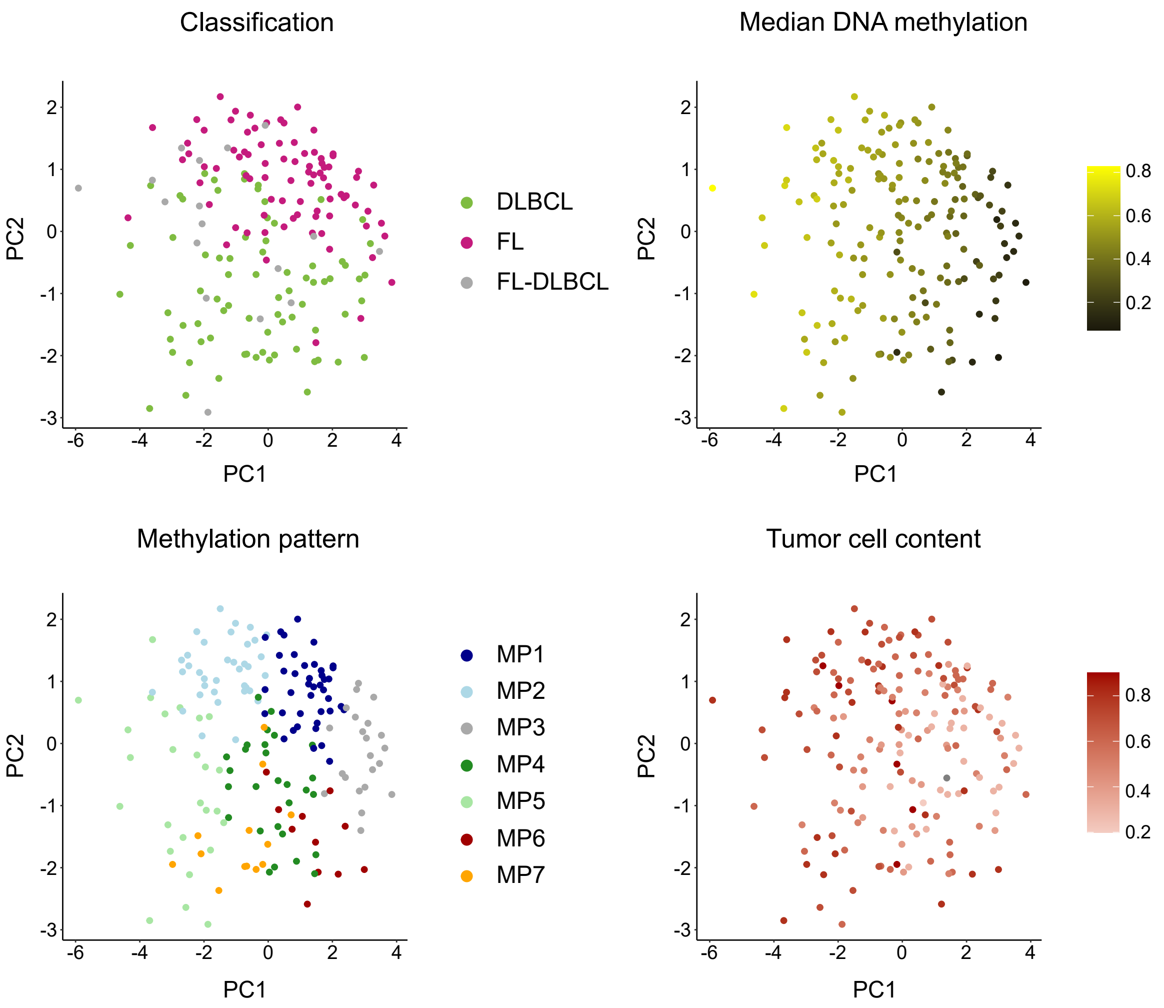

Supplementary Figure S7: Principal component analysis (PCA) of the 300 CpGs in germinal center-derived B-cell lymphomas. Plots are colored according to various features: lymphoma classification, median DNA methylation levels, methylation patterns (MPs) and tumor cell content based on whole genome sequencing. FL: Follicular lymphoma; DLBCL: Diffuse large B-cell lymphoma.

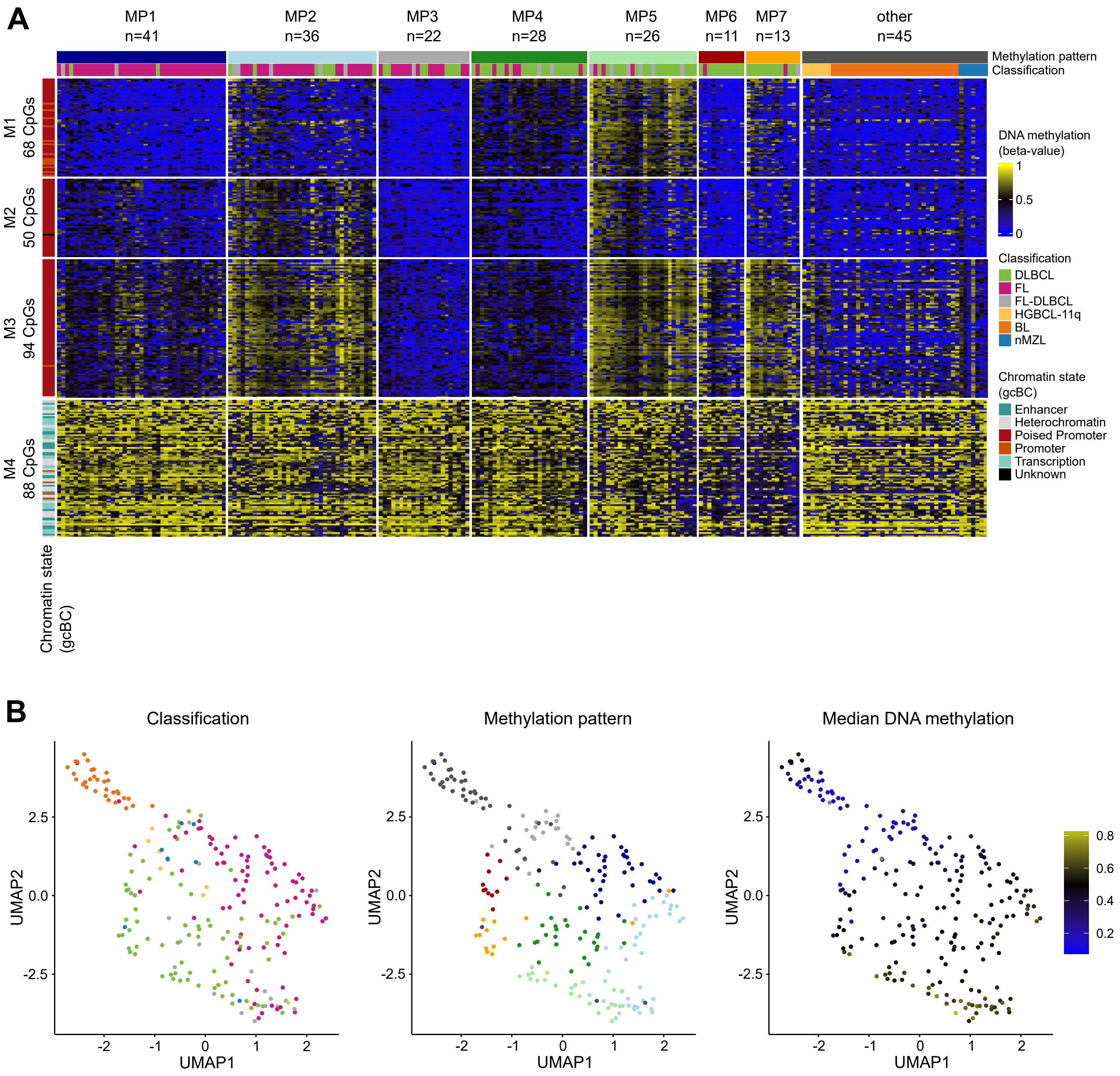

Supplementary Figure S8: DNA methylation profiling of the 300 CpGs across various B-cell lymphoma entities. A: Heatmap illustrating DNA methylation levels of the 300 CpGs as shown in Figure 1 with additional B-cell lymphoma subtypes ("other") displayed on the right side, including HGBCL-11q (n=7), BL (n=31) and nMZL (n=7) (López et al, 2019; Kretzmer et al., 2015; Loeffler-Wirth et al., 2022). Rows represent individual CpGs, and columns represent samples. **B:** UMAP analysis (Manhattan distance, 15 neighbors) based on the 300 CpGs. Plots are colored according to lymphoma classification, methylation patterns, and median DNA methylation levels. FL: Follicular lymphoma; DLBCL: Diffuse large B-cell lymphoma; HGBCL-11q: High-grade B-cell lymphoma with 11q aberrations; BL: Burkitt lymphoma; nMZL: nodal marginal zone lymphoma.

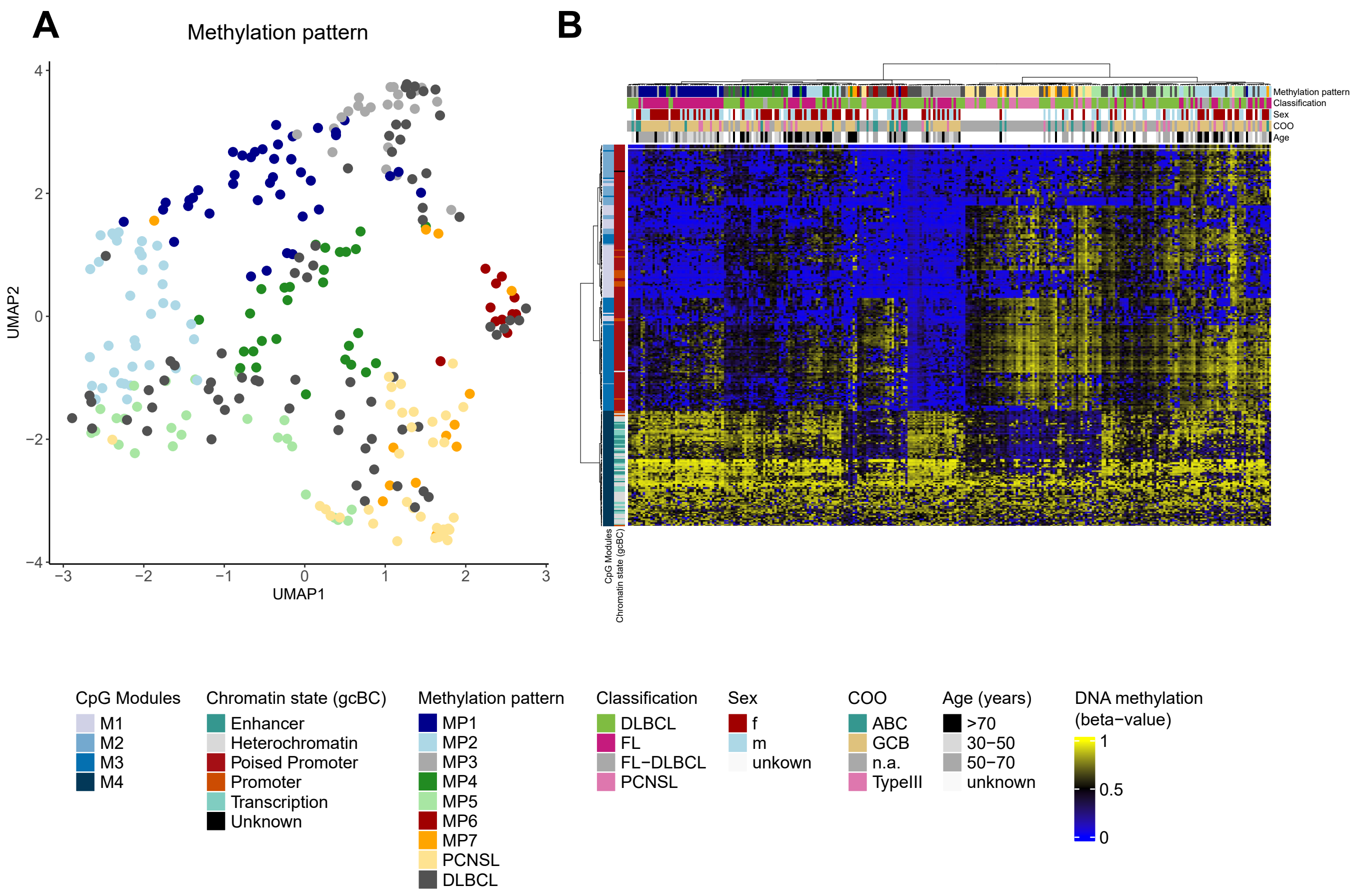

Supplementary Figure S9: DNA methylation profiling of the 300 CpGs across other DLBCLs and PCNSLs. A: UMAP analysis (Manhattan distance, 15 neighbors) based on the 300 CpGs including additional DLBCL cases (n=69, Carlund et al.) and PCNSL (n=34, Carlund et al. and Vogt et al.). **B:** Heatmap illustrating DNA methylation levels of the 300 CpGs as shown in Figure 1 with additional B-cell lymphomas. Rows represent individual CpGs, and columns represent samples. FL: Follicular lymphoma; DLBCL: Diffuse large B-cell lymphoma; PCNSL: Primary central nervous system lymphoma.

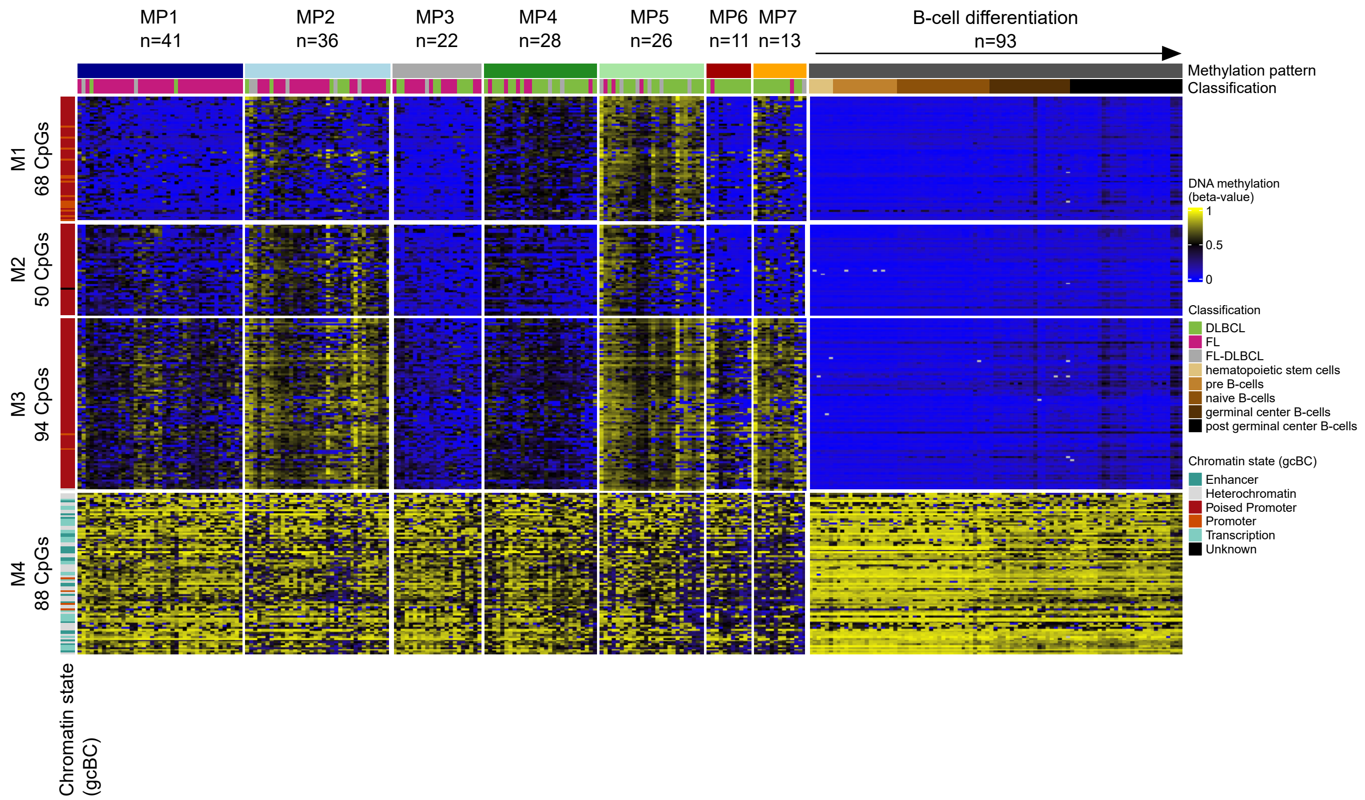

Supplementary Figure S10: DNA methylation profiling of the 300 CpGs across non-malignant (pre-)B-cell subpopulations. Heatmap illustrating DNA methylation levels of the 300 CpGs as shown in Figure 1 with non-malignant (pre-)B-cell subpopulations displayed on the right side. Rows represent individual CpGs, and columns represent samples. B-cell subpopulations are organized according their differentiation state. FL: Follicular lymphoma; DLBCL: Diffuse large B-cell lymphoma.

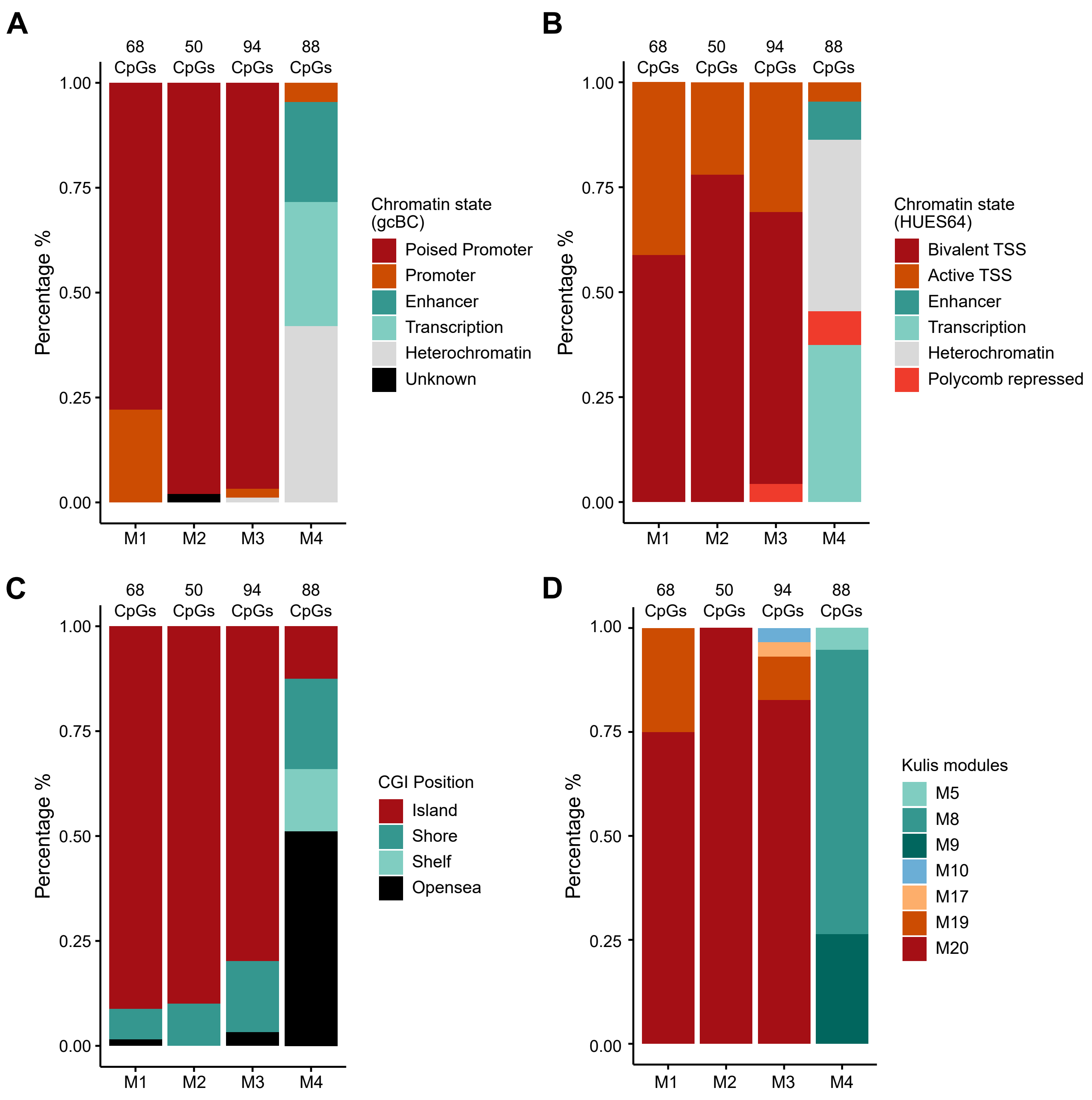

Supplementary Figure S11: Genomic feature localization of the 300 CpGs. Bar plots illustrate the distribution of these CpGs within chromatin states defined in germinal center-derived B-cells (A), chromatin states defined in the human embryonic stem cell HUES64 (B), their positions relative to CpG islands (CGIs, C) and within the dynamic modules defined by Kulis et al. (D). Statistical comparisons are summarized in Supplementary Table S5. Shelf: ~ 4 Kb from islands; Shore: ~ 2 Kb from islands; TSS: Transcription start site.

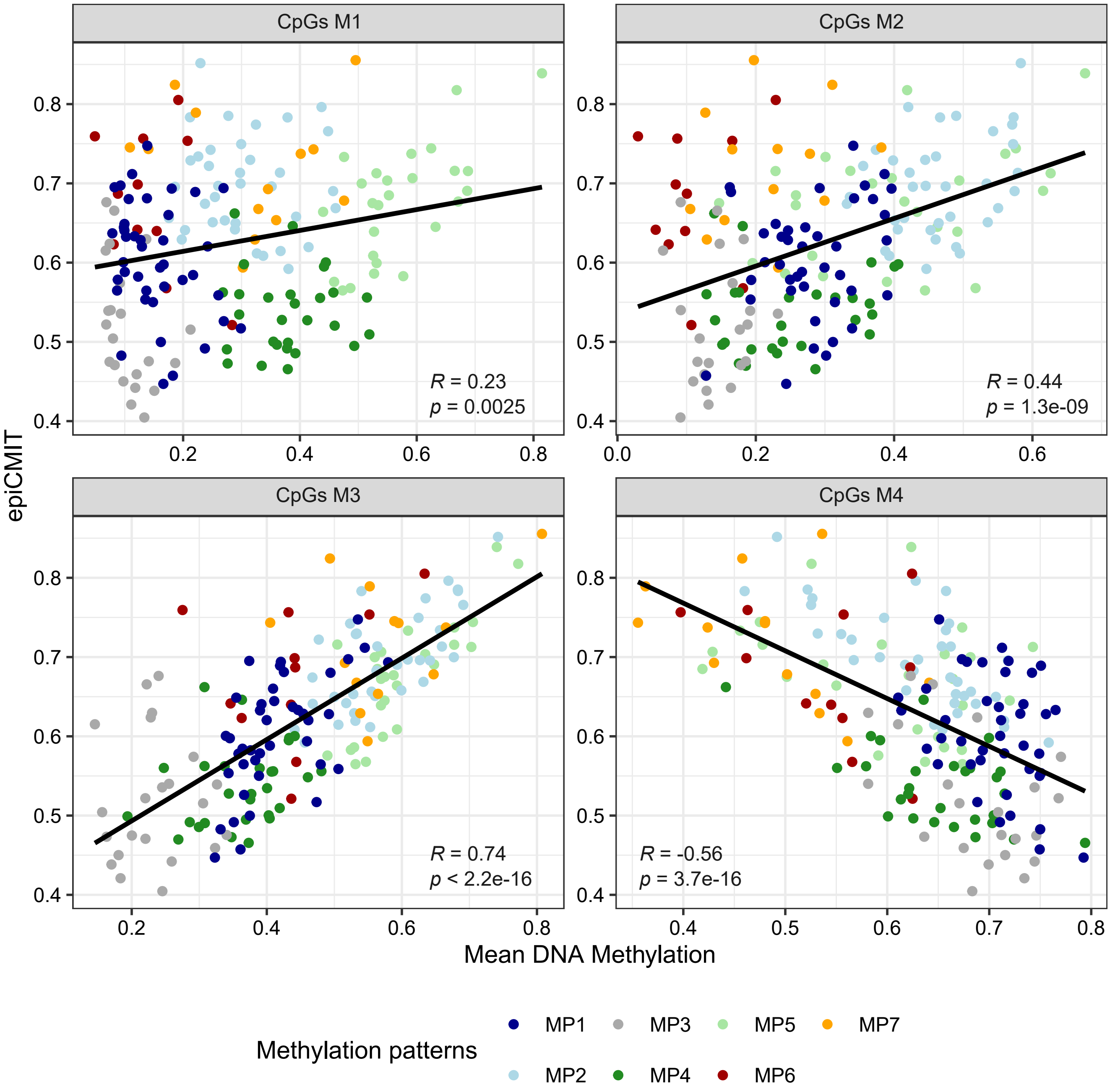

Supplementary Figure S12: Correlation of the mean DNA methylation against the proliferation history (epiCMIT). Mean DNA methylation for each sample across each CpG module (M1-4) was calculated and correlated against the proliferation history, assessed through the epiCMIT package. Pearson correlation analysis was performed.
